# A Practical Treatise on Fractures and Dislocations

**Published:** 1899-07

**Authors:** 


A PRACTICAL TREATISE ON FRACTURES AND DISLOCATIONS. By LEWIS A. STIMSON, B. A., M. D., Professor of Surgery in Cornell University Medical College; Surgeon to New York and Hudson Street Hospitals; Consulting Surgeon to Bellevue, St. Johns and Christ Hospitals. Octavo, 823 pages, 326 illustrations, 20 plates. Philadelphia: Lea Brothers & Co. 1899.

The great trouble which has been most apparent in books on special subjects such as this one has been a lack of meatiness. This was true, in a measure, of the first and second editions of this work which seemed, however, to be more or less of a necessity as a valuable adjunct to medical literature. The first editions were rather more of history than practice, if one judges them from a purely critical viewpoint. They were published in 1883 and 1888. Since then Dr. Stimson has gone over the field most thoroughly and has now given the profession a book which is of distinct value in every-day life. Strange as it may seem the fact remains, nevertheless, that surgeons are constantly running across cases of fracture and dislocation which have been put up improperly by general practitioners. Fractures and dislocations are looked upon by too many men as merely passing events in the everyday life of professional work. The interest, the importance and the dangers are overlooked. A careful reading of Dr. Stimson's work will do much to correct this, and a doctor who erred after such a reading would better take down his sign and enter upon a pursuit where intelligence is a convenience, but not an absolute necessity.

There is less history in this edition and more practice. Rarer forms of fractures and dislocations—and rare as some of them are, one is liable to meet with them at any time—are given ample space. The section on fractures in general has been almost entirely rewritten, and there is a new classification and arrangement of skull injuries. The operative reduction of dislocations is handled with the skill one would expect from Dr. Stimson. Many of the conditions treated of are illustrated with skiograms; and, en passant, Dr. Stimson has an *x*-ray opinion which is interesting. He says: "While the *x*-rays have been of interest and value in showing details of certain fractures, especially at the wrist, elbow and ankle, yet it cannot fairly be said that they have yielded much information of practical value which could not have been obtained by palpation. Probably their influence will be increased by improvements in methods and apparatus, but at present the information which they give needs to be sifted with great care from among many misleading appearances."

Every fracture possible is treated of at length, and pathology, etiology, symptoms, diagnosis, treatment and repair are set before the reader in a most entertaining and instructive manner. Complications and remote consequences, delayed union, failure of union, and faulty union are all given careful consideration.

The same general plan is followed in handling dislocations in general, special stress being laid upon accidents that might be caused by attempting to reduce dislocations; congenital dislocations and spontaneous dislocations are also well set forth.

The publishers have given the work most excellent and generous treatment in the way of letter press, binding and illustrating. As the book stands now, it is well-nigh perfect in all respects.

